# Subcutaneous Immunization of Dogs With *Bordetella bronchiseptica* Bacterial Ghost Vaccine

**DOI:** 10.3389/fimmu.2019.01377

**Published:** 2019-06-25

**Authors:** Abbas Muhammad, Johannes Kassmannhuber, Mascha Rauscher, Alaric A. Falcon, David W. Wheeler, Alan A. Zhang, Petra Lubitz, Werner Lubitz

**Affiliations:** ^1^BIRD-C GmbH & Co KG, Vienna, Austria; ^2^Centre of Molecular Biology, University of Vienna, Vienna, Austria; ^3^ELANCO Animal Health, Greenfield, IN, United States

**Keywords:** *Bordetella*, Bacterial Ghosts (BGs), whooping cough, immunization (vaccination), dog model

## Abstract

The *Bordetella* species are Gram-negative bacterial pathogens that colonizes mammalian respiratory tract causing respiratory diseases in humans and animals. *B. bronchiseptica* causes clinical conditions in many mammals including immunocompromised humans. Using the dog model of respiratory infection, it has been shown in this study that a newly developed *B. bronchiseptica* Bacterial Ghost (BbBG) vaccine exhibited significant protection in the face of a severe pathogenic bacterial challenge in seronegative dogs. The protein *E*-specific lysis mechanism was used to produce BbBGs. Bacterial Ghosts (BGs) are the empty cell envelope of Gram-negative bacterium. They are genetically processed to form a microscopic hole in their membrane, through which all the cytoplasmic contents are expelled leaving behind intact empty bacterial shells. Due to the intact surface structures of BGs, they offer the safety of inactivated but efficacy of live attenuated vaccines. In this study, seronegative dogs were vaccinated subcutaneously (s/c) with two different doses of a newly developed BbBG vaccine [lower 10^∧^5 (BbBG – 5) and higher 10^∧^7 (BbBG – 7)] on day 0 and 21. The animals were challenged (by aerosol) with virulent live *B. bronchiseptica* strains 41 days after first vaccination. The dogs vaccinated s/c with BbBG – 7 vaccine had significantly lower spontaneous coughing scores (*P* = 0.0001) than dogs in negative control group. Furthermore, the tested BbBG – 7 vaccine was equivalent to the positive control vaccine Bronchicine CAe in terms of safety and efficacy. For the first time, we report the successful use of liquid formulated BGs vaccines in animal studies. Earlier reported studies using BGs vaccines were performed with resuspended freeze-dried BGs preparations.

## Introduction

The *Bordetella bronchiseptica* is gram-negative aerobic coccobacillus and is widely known for causing canine infectious respiratory disease (CIRD) also known as kennel cough. Apart from dogs, *B. bronchiseptica* causes respiratory disease in wide range of mammals including immune-deficient humans (1, 2) and in individuals with history of close contact with infected dogs or dogs that are recently vaccinated with live attenuated *B. bronchiseptica* vaccine (3–8). It causes snuffles in rabbits, pneumonia in guinea-pigs and atrophic rhinitis in swine (3, 4, 9–15). *B. bronchiseptica* infection is endemic in many non-human mammalian populations, and a particular high incidence of infections is seen in kennels as well as pig farms, where extensive vaccination is used to prevent disease (15, 16). Several whole cell bacterin (17), antigen extract (18), and modified-live vaccines (19–21) have been successfully tested in dogs via intranasal (IN) route of administration. *B. bronchiseptica* colonizes the ciliated respiratory epithelium of dogs and cats and is not found in other body tissues (22). Due to its colonization, the canine respiratory cilia lose their beating motion within 3 h of a phase I or an intermediate phase *B. bronchiseptica* infection where almost 100% of ciliary activity is lost (23). Due to this ciliostasis, the respiratory epithelia of dogs are more prone to secondary viral and bacterial infections (22–24).

The Bacterial Ghost (BG) system is an advanced approach for the production of safe and potent vaccines in the prevention and control of a wide range of infectious diseases (25–27). BGs are produced by expression of cloned gene *E* of bacteriophage ΦX174 under tight expressional regulation (25, 26, 28–32). Protein *E* expression initiates the formation of a trans-membrane tunnel structure spanning the whole cell envelope, through which the entire cytoplasmic content is expelled. This expulsion of cytoplasmic content is due to the difference in osmotic pressures between the cell interior and the culture medium (33). These resulting empty bacterial cells have a wide range of use, as a vaccine or a delivery vehicle for transporting other immunogens or biologically active substances (34, 35). BGs have the advantage over other inactivated non-living vaccines in terms of efficacy as all of the surface structural components of the BG envelope are non-denatured and remain intact (36). The process of producing BGs is gentle and does not harm the essential structural components of the bacteria. The resulting particles are immunologically active and are capable of stimulating the host immune system. Further, they can deliver recombinant antigens (Ag) to professional antigen presenting cells (APCs) through Toll-like and pattern-recognition receptors, making them ideal for parenteral and mucosal administration (32, 37–39). Recombinant foreign antigens can be used in conjunction with BGs in several ways. They can be incorporated into BGs and displayed on the BGs surface. The BGs can also carry recombinant antigen within its inner lumen. Finally BGs and recombinant antigens can be simply mixed together, to utilize the intrinsic adjuvant effect of BGs (40–42). Production of BGs is an efficient, stable and safe process resulting in freeze dried vaccine preparations which are stable at ambient temperatures for many months (43); and for the first time as shown in this communication, BGs are stable in liquid formulation for several months.

There are several *Bordetella* vaccines available on the market. These vaccines are administered via different routes and have different protection levels. Most veterinarians prefer injectable vaccines over oral or intranasal formulations due to its ease of administration in vicious and difficult to control animals. That is why there is always a need for better, safe, and efficient injectable vaccine. In present study, we evaluated the protection conferred by an injectable vaccine which is a cell antigen extract of *B. bronchiseptica* vs. a more defined envelope of *B. bronchiseptica* produced using propriety Bacterial Ghost platform technology.

## Materials and Methods

### Ethical Statement and Animal Welfare

The animal study was performed in accordance with the Guide for the Care and Use of Laboratory Animals (Eighth Edition, 2011) of National Research Council Academies. The National Academies Press, Washington DC, Title 9 Code of Federal Regulations, Part 103.3. The protocol was reviewed and approved by Institutional Animal Care and Use Committee of Ridglan Farms (no. BIOUS140044). This study was conducted to test efficacy of novel experimental biological products (vaccines) and did not represent an unnecessary duplication of research.

### Bacterial Strain, Plasmids, and Growth Conditions

*B. bronchiseptica* strain 110H dog isolate was obtained directly from David Bemis, at the University of Tennessee. The bacteria were grown on Tryptose Phosphate Agar (TPA) (Difco Laboratories, US) and or on Bordet Gengou Agar plates (BGA) (Difco Laboratories US) supplemented with 15% defibrinated sheep blood (VWR international—ROCKR111-0050) and incubated at 36°C for 24–36 h. For bacterial transformation lysis plasmid pGLysivb (43) was used in which the expression of lysis gene *E* is driven by the λPR_*mut*_ – c*I*857 promoter-repressor system to regulate the lysis gene *E* expression by temperature up-shift from 36 to 42°C. Plasmid pBBR1MCS-5 (44) is a broad host range cloning vector and was used for construction of lysis plasmid pGLysivb. Plasmid pBBR1MCS-5 lacking the lysis gene *E* was used for control experiments. For bacterial selection, gentamycin was used at final concentration of 20 μg/mL. Both of the above mentioned plasmid DNA were isolated from *E. coli* C2988J using Pure Yield Plasmid Midiprep system (Promega) using the manufacturer's protocol. The identity of the obtained plasmid DNA was confirmed by restriction enzyme analysis using FastDigest® restriction enzymes (Fermentas). The purity and quantity of plasmid DNA was evaluated using NanoDrop ND-2000 (Peqlab).

### Electro Competent Cell Preparation and Transformation

The electroporation experiments were performed as described by Miller et al. (45) with slight modifications. For production of competent cells, *B. bronchiseptica* 110H was grown on Bordet Gengou Agar plates (supplemented with 15% defibrinated sheep blood) for ~16 h at 36°C after which the cells were harvested in ice cold GlySuc buffer (15% Glycerol and 272 mM Sucrose solution) and pelleted at 5,000 × g for 10 min at +4°C. The cells were gently resuspended and washed in half volume of GlySuc buffer (same conditions as above) followed by wash with quarter (¼) volume of GlySuc buffer. Cells were resuspended gently in 300–500 μL of GlySuc buffer (depending on the size of pellet) and aliquoted in ~100 μL to be used immediately for electroporation or stored at −80°C for later use. One to three microgram of isolated plasmid DNA (pGLysivb or pBBR1MCS-5) was added to the 100 μL aliquot of competent cells and the mixture was incubated on ice for 45–60 min. High voltage pulses were delivered to the ice cold samples that are shifted to 1 mm gap cuvettes (Peqlab). Genepulser® II, Electroporation system (Bio-Rad) was used with following settings: 2.5 Kv, 25 F, 400 Ω with time constraint ranging from 3.5 to 7 ms. Following the electroporation the cells were regenerated by addition of 800 μL of Tryptose Phosphate vegetable source (TPv) broth (Difco Laboratories) and incubated at 34°C for 90 min. The use of TPA is described in EMA (46). This media is used in later development and in final stage of vaccine production as needed by United States Department of Agriculture (USDA) regulation which restricts the use of blood agar in production of veterinary vaccines. The regenerated cells were then plated on TP-agar plates (Difco Laboratories), supplemented with 20 μg/mL of gentamycin. Screening of positive recombinant strains was performed via restriction enzyme digestion (Fermentas).

### *Bordetella bronchiseptica* Bacterial Ghost Production

Cells were grown in Tryptose Phosphate broth—vegetable source (TPv) (Difco Laboratories), supplemented with 0.004 g/L nicotinic acid, 0.02 g/L FeSO_4_7.H_2_O, and 0.02 g/L ascorbic acid. The medium scale fermentation of *B. bronchiseptica* 110H were performed in Labfors 3 fermenter (Infors Ag, Bottmingen, Switzerland) with working volume of 4 L. Medium scale fermentation was performed in TPv supplemented with 0.004 g/L nicotinic acid, 0.02 g/L FeSO_4_7.H_2_O, and 0.02 g/L ascorbic acid and gentamycin at 20 mg/L. Five milliliter of 30 h culture of *B. bronchiseptica* 110H (carrying lysis plasmid pGLysivb) was used to prepare 200 mL pre-culture (35°C for 12–14 h). This pre-culture with the OD_600nm_ of 1.15 was used to inoculate the fermenter containing 3.8 L of TPv to reach initial starting OD_600nm_ of 0.052. The cells were grown to OD_600_ of ~0.5 at 35°C and the lysis was induced by temperature upshift to 44°C. The lysis was complete after 420 min after which the lysed cells were washed to get rid of the cytoplasmic contents using 4 L of sterile dH_2_O using Tangential Flow Filtration (TFF) module (0.2 μm cut-off, GE healthcare) for 60 min. This was accomplished by matching the flow of media out of the TFF module with the same amount of dH_2_O pumped into the fermenter. During the course of fermentation, following parameters were documented: temperature, flow, stirrer, pH, pO_2_, foaming, and pumps for acid and base. Antifoam-A (Sigma) was added via sterile septum when needed. After the completion of washing, the broth was concentrated to approximately 200 mL in TFF module (0.2 μm cut, GE, healthcare) and flushed from the module with ~200 mL sterile dH_2_O into the “inactivation bottle” to get a final volume of ~400 mL (conc. I).

### Final Inactivation and Vaccine Storage

Due to regulatory requirements for veterinary vaccines by United States Department of Agriculture (USDA) and European Medicines Agency (EMA), the remaining un-lysed cells or escape mutants are inactivated/killed by one of the prescribed chemical procedures (47). To fulfill the above requirement, ethylenimine (EI) was used. A final concentration of 20 mM EI (FERAK, Berlin) was added to conc. I and incubated for 3 h with slight agitation at 35°C (2 mM, if calculated for initial volume of 4 L before concentration). Samples for *cfu* count and microscopy were drawn hourly and the inactivation activity was halted by addition of 1 M sodium thiosulfate, at 10% of initial volume of EI used and kept for another 30 min at 35°C, while shaking. The inactivated sample was washed with 4 L of sterile dH_2_O using a fresh TFF module (sterile 0.2 μm cut-off, GE, healthcare). This was accomplished by matching the flow of media out of the TFF module, with the same amount of dH_2_O pumped into the inactivation bottle. Later the broth was concentrated to ~200 mL, before being flushed out of the module with ~200 mL sterile dH_2_O to bring the volume to ~400 mL (conc. II). This sample was stored at −20°C for ~15 days, and was later thawed, and further diluted in dH_2_O for final dose defining and shipped in liquid form at +4°C, for animal experiments. The doses were adjusted by performing flow cytometry as discussed in Langemann et al. (43). In the current study, the lysis efficiency of gene E was calculated to be 98% and remaining bacteria are inactivated by EI as described above. Based on the calculations from flow cytometry, two in 100 part of vaccine contains killed bacteria. The final highest dose contains 20 k of killed bacteria compared to ~1 Million BbBGs. The animals were vaccinated with BbBG vaccine approximately after 3 months of liquid formulation which was shipped and stored at +4°C upon arrival.

### Animals and Animal Housing

Thirty-two *B. bronchiseptica* seronegative dogs (beagles 7 weeks old) were selected and housed in the research facility at Ridglan Farms for the vaccination phase (8 weeks old) and later at BSL-2 facility of the University of Wisconsin for the challenge phase (14 weeks old). Blood samples from all animals were collected prior to inclusion in the study. Serum was separated from the blood and used to determine antibody titers against *B. bronchiseptica*. The levels of anti-Bb antibodies in the blood of each dog was determined by enzyme-linked immunosorbent assay (ELISA). Seronegative dogs had an ELISA titer ≤ 20 as described in Dees et al. (48). The study animals were randomly assigned to one of the treatment groups—T01–T04 with (*n* = 8) animals per treatment group. The randomization was performed by the study statistician. Animals in each group were assigned unique identification codes and ear tattooed for easy record. The puppies in different treatment groups were three-housed together in stainless steel caging at Ridglan Farms during vaccination phase and gang housed during the challenge phase at (bio safety level-2) BSL-2 facility of University of Wisconsin as prescribed in “*Guide for care and Use of Laboratory Animals*” by regulatory bodies (2001). Animals were fed with high density canine diet 5L18 PMI nutrition international LLC or similar quality diet and had access to water *ad libitum* in both facilities. Since *B. bronchiseptica* is infectious bacteria, strict isolation procedures were used during the challenge phase at University of Wisconsin in BSL-2 facility.

### Study Design and Study Vaccine

The dogs were moved to the vaccination facility at Ridglan farms on study day−7, study animals were observed daily to determine general health status by qualified veterinarian to ensure that the subjects are free of any kind of respiratory diseases. Observations were made at approximately the same time each day and were documented on the Health Observation Record. Animal health observations consisted of cage side visual assessments of animals for indicators of animal health. Since the shedding of *Bordetella* is of epidemiological interest in this disease the nasal swabs were collected at day−1 and 20 from all dogs before the 1st and 2nd vaccinations occurred respectively, and on day 41 through 44, 46, 48, 52, and 54. Two swabs were collected from each dog, one from each nostril. The swab samples were used for quantitative PCR and *B. bronchiseptica* culturing which are available as a diagnostic service at the university of Wisconsin – Madison – Wisconsin Veterinary Diagnostic laboratory. Similarly, serum samples were used to monitor *B. bronchiseptica* titers. Blood samples were collected from jugular/cephalic vein in 4 mL serum separation tube (SST) before each vaccination; day−1 and 20, before challenge; day 41 and at the end of study; day 54.

Group—T01 dogs were used as negative control (PBS). Group—T02 dogs were used as positive control and vaccinated with commercially available vaccine—Bronchicine CAe. Group—T03 dogs were vaccinated with 1 × 10^7^ *B. bronchiseptica* Bacterial Ghosts (BbBG – 7) and Group—T04 dogs were vaccinated with 1 × 10^5^ *B. bronchiseptica* Bacterial Ghosts (BbBG – 5) (Table 1). All animals were injected with 1 mL of respective test material. Vaccines were administered on day 0 and again on day 21. All vaccines were administered subcutaneously in the interscapular region (between the shoulder blades at the base of neck). First vaccination was on the right side, and the second vaccination was given on the left side. A patch of hair was clipped prior to vaccination at the injection site so that it was easier to examine the dogs for injection site reactions. Latex examination gloves were worn by all personnel during the vaccination. Gloves were changed regularly after handling each dog to avoid chances of cross contamination. Dogs were monitored for systemic and or local injection site reactions after each vaccination. Examinations occurred at ~4, ~24, ~48, ~72 h post vaccination. Monitoring consisted of mainly body temperature, general attitude/behavior, and for injection site reactions in dogs. For reactions present at the 3 day post-vaccination examination, monitoring was continued daily until the reaction was resolved.

**Table 1:** Study design and groups.

**Treatment group**	**Experimental biological product and (estimated dose)**	**No. of animals**	**Route of administration**	**Challenge**
T01	PBS (Negative control)	8	s/c	*B. bronchiseptica*
T02	Bronchicine CAe (Positive control)	8	s/c	*B. bronchiseptica*
T03	BbBG – 7 (~1 × 10^7^ BG particles)	8	s/c	*B. bronchiseptica*
T04	BbBG – 5 (~1 × 10^5^ BG particles)	8	s/c	*B. bronchiseptica*

*PBS, phosphate buffer saline; BbBG, B. bronchiseptica Bacterial Ghosts; s/c, sub cutaneous*.

### Challenge and Clinical Observations

All dogs were challenged intranasally with a mixture of 2 strains of virulent *B. bronchiseptica* on day 42 after the 1st vaccination. Strains 87 and 110H were provided by Dr. David Bemis (University of Tennesse) and were grown in the laboratory of Dr. Ronald D. Schultz (University of Wisconsin). The challenge cultures were prepared at the challenge facility by thawing and plating bacteria onto blood agar plates. The bacteria was allowed to grow at 37°C. *B. bronchiseptica* was then harvested by scraping cells from the plates and suspending both cultures in tryptose phosphate media at a target of 1.0 × 10^∧^10 cfu/mL for each culture. The dogs were exposed four at a time to the challenge (aerosolized *Bordetella*) for 20 min in an isolator cage. The challenge dose was ~10 mL at target concentration of 1 × 10^8^ CFU/m^3^. Nebulizer was used for ~15–20 min in 1 m^3^ isolator cage. Following challenge the remaining mixed challenge material was tittered at ~2.2 × 10^∧^10 cfu/mL. All clinical observations were blinded to the treatment group. Baseline challenge clinical observations were made on study day 42, prior to challenge. Like in the majority (49) of studies, a scoring system was used to asses clinical signs after challenge. The study animals were observed daily after challenge beginning on study day 43 and continuing through study day 54. These observations consisted of 20 min in-room visual assessments of animals for clinical signs of B. bronchiseptica induced tracheobronchitis. Clinical signs included, but were not limited to, the following: the number of spontaneous coughs, retching, +/− labored breathing. At the conclusion of the 20 min observation period, each dog was observed for nasal and conjunctival discharge. Labored breathing was noted if present. After which animals were administered mild laryngeal palpation for induced coughing signs. This consisted of placing a thumb and forefinger on either side of the larynx with sufficient pressure to move the skin up and down with the palpation, without causing undue pressure.

### Bacteriologic Culture

To quantitate growth of *B. bronchiseptica* in swab samples, selective culture methods (50) and standard semi-quantitative techniques were used (51). Briefly, swab specimens from the nasal cavity were inoculated onto 1 quadrant of Mc-Conkey and peptone agar plates. A sterile bacteriologic loop was then used to sequentially streak the 4 quadrants of each plate without flaming the loop until all quadrants were streaked. Plates were incubated at 37°C and checked at 24 and 48 h for growth of microorganisms. Suspicious colonies (i.e., organisms that grew as non-lactose fermenters on Mc-Conkey's agar and as blue colonies on peptone agar) were quantitated as follows: no growth, 0; growth on the first quadrant, 1 +; growth in first and second quadrants, 2+; growth in first 3 quadrants, 3+; growth in all quadrants,4+ Data derived from peptone agar (plate counts) were analyzed statistically. To confirm that quantitated colonies were *B. bronchiseptica*, typical colonies were sub-cultured on blood agar and tested by use of conventional methods of identification. TSI (alkaline), urease (+), citrate (+) and arginine (−) were used as a positive confirmation of *B. bronchiseptica*.

### Statistical Analysis

The individual animal was evaluated as the experimental unit. The primary variable was spontaneous coughing. Each animal was given a spontaneous coughing score according the number of coughs in the 20 min observation time: 0 = coughing absent, 1 = occasional cough (1–2 coughs), 2 = frequent (≥3 coughs). Secondary variables included the other clinical signs of *Bordetella* infection: laryngeal palpations, nasal discharge, and ocular discharge. These were also place in a scoring system. Laryngeal palpations were scored by 0 = little to no cough, 1 = prolonged cough, 2 = severe coughing. Nasal and ocular discharge were similarly scored with 0 = Normal, 1 = clear discharge, 2 = mucopurulent. Differences were evaluated using two-sided tests at alpha = 0.1. For each dog, the severity spontaneous of coughing (maximum coughing score of 2 post-challenge), the duration of spontaneous coughing (number of days with coughing scores of 1 or 2), and the summed spontaneous coughing scores during the post-challenge were three methods of evaluating the primary outcome variable. The maximum score for coughing and the summed coughing scores were evaluated as a categorical variable. The probability that an observation from a vaccination group was different from an observation from the control group (or that there was a shift in distribution between the groups) was tested using Wilcoxon's Rank Sum Test (the NPAR1WAY procedure in SAS, SAS Institute, Cary NC, SAS/STAT 13.1). Maximum scores and summed scores were analyzed using mitigated fraction (MF) and a 95% confidence interval calculated (CI; the FREQ procedure in SAS). The influence of vaccination on the duration of coughing was evaluated using Wilcoxon's Rank Sum Test (the FREQ procedure in SAS). The placebo control and positive control were compared to each of the remaining groups. Experimental biological products were also compared to each other.

Least squares means and standard errors by treatment group (over time where appropriate) are used to summarize the results. Counts and frequencies are used as appropriate. Arithmetic means and standard deviations by treatment group over time are also provided. Clinical scores for coughing, ocular discharge, nasal discharge, and laryngeal palpation were analyzed individually and as a composite score. They were statistically analyzed as ordinal data using Wilcoxon's Rank Sum Test (the NPAR1WAY procedure in SAS). Results from each day were analyzed independently. If within day treatment effects were significant, the placebo control and positive control were compared to each of the remaining groups; differences between groups were evaluated using an unadjusted alpha = 0.1. Median scores by day are used to summarize the results.

### Special Test Criteria

The study was to be considered valid if all animals were sero-negative for *B. bronchiseptica* prior to entry into the study. Dogs were also to remain seronegative prior to vaccination, and the negative controls must be seronegative prior to challenge. These criteria were met for the study. Further the test was to be considered invalid if more than 2 of the control dogs showed no typical signs of *Bordetella* infection after challenge. This was a valid test with all control dogs showing signs of infection.

## Results

### *Bordetella bronchiseptica* Bacterial Ghost Vaccine Production

**Figure 1:**
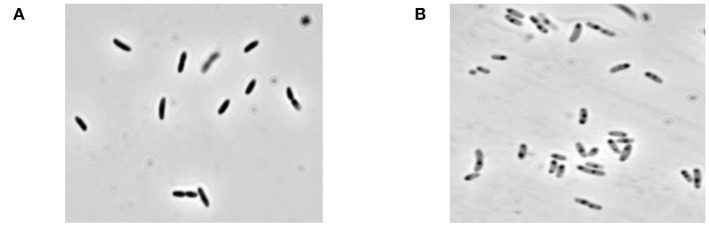
Microscopic picture of *B. bronchiseptica* Bacterial Ghosts (BbBG). **(A)** *B. bronchiseptica* whole cell (before lysis). **(B)** *B. bronchiseptica* BGs (end of lysis and after inactivation with EI).

Lysis plasmid pGLysivb (43) was used to transform the *B. bronchiseptica* strain 110H. The plasmid pGLysivb allowed the induction of gene *E* by a temperature upshift of 36–44°C. Transformed *B. bronchiseptica* 110H was grown with aeration in a total volume of 4 L, and the OD and live cell counts were monitored during the growth and lysis phase. The lysis was continued to 420 min after which the cells were washed with 4 L of dH_2_O using TFF and harvested. The CFU count showed a lysis efficiency of 98% and the phase contrast microscopy showed a typical empty cell appearance as seen in the most BG preparations (Figure 1). The remaining survivors were killed by incubating the culture harvest with 20 mM of Ethylenimine solution at 35°C for 3 h and cells were once again washed with 4 L dH_2_O and concentrated to 400 mL using TFF. No single bacterial colony was detected on the plates with EI treated samples incubated at 36°C for up to 7 days.

In this study, freeze-dried BGs have been substituted by a liquid formulation. After production, BbBGs were stored at −20°C (~2 weeks). The vaccine was later thawed, reconstituted in dH_2_O for final dosage and stored at +4°C until it was used for animal testing. A portion of this liquid formulation of BbBG vaccine was used for monitoring vaccine stability through microscopy and FACS analysis and was found to be stable for several months at +4°C. This procedure was novel for BG vaccines and mimics veterinary practice to store vaccines in liquid form which is ready to use. The BbBG sample was stored at +4°C until administration. A portion of the final BG preparation was used for sterility testing. No bacterial or fungal growth was observed after 14 days of incubation in Tryptic Soy Broth enrichment medium at +36°C.

### Vaccine Efficacy and Safety

**Figure 2:**
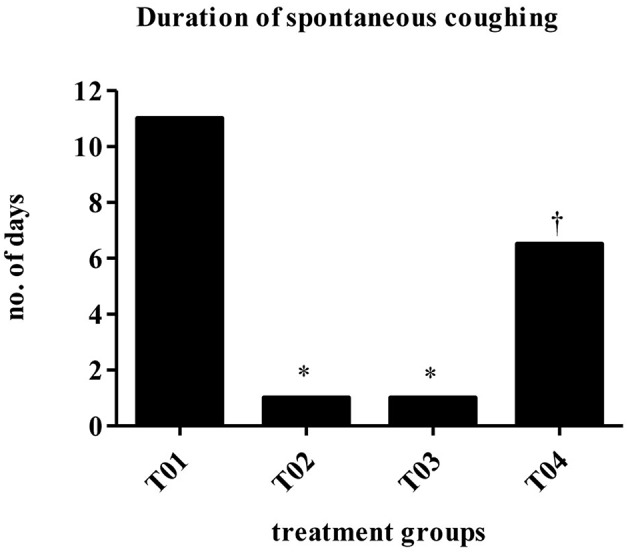
Duration of spontaneous coughing (median no of days). Overall treatment *P* = 0.0003. ^*^vs. T01, *P* < 0.10. ^†^vs. T02, *P* < 0.10. T01, PBS control. T02, Bronchicine CAe. T03, *B. bronchiseptica* Bacterial Ghosts (1 x 10^∧^7 cells/mL). T04, *B. bronchiseptica* Bacterial Ghosts (1 × 10^∧^5 cells/mL).

**Figure 3:**
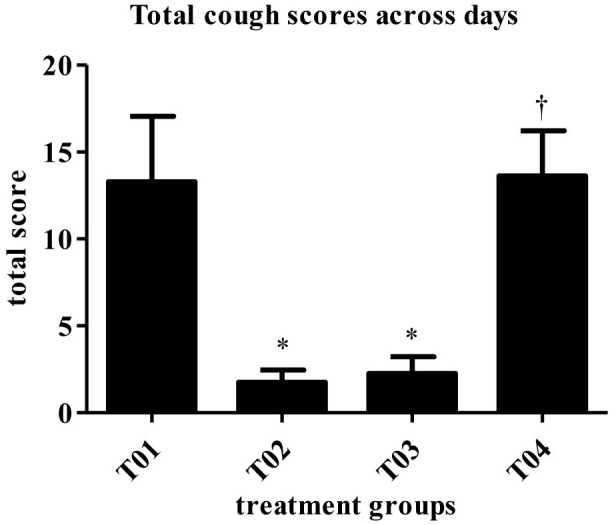
Score totaled across days (mean). Overall treatment *P* = 0.0001. ^*^vs. T01, *P* < 0.10. ^†^vs. T02, *P* < 0.10. T01, PBS control. T02, Bronchicine CAe. T03, *B. bronchiseptica* Bacterial Ghosts (1 × 10^∧^7 cells/mL). T04, *B. bronchiseptica* Bacterial Ghosts (1 × 10^∧^5 cells/mL).

Dogs vaccinated s/c with newly developed high dose BbBG – 7 vaccine and commercially available *Bordetella* vaccine—Bronchicine CAe demonstrated significant protection from spontaneous coughing, duration of coughing and induced coughing scores when compared to placebo treated dogs. The median number of days of spontaneous coughing was significantly affected by treatment (*P* = 0.0003). Dogs in groups—T02 and T03 had significantly fewer days of coughing than dogs in group—T01 (control group). Dogs in group—T04 had more days of coughing than dogs in group—T02 and was not statistically different than group—T01 (Figure 2). Mean post-challenge cough scores were significantly lower (i.e., coughing was less severe) in the groups T02 (vaccinated with Bronchicine CAe) and T03 (vaccinated with BbBG – 7) compared to groups T01 (placebo control) and T04 (vaccinated with BbBG – 5) (*P* > 0.005). Cough scores in low dose BbBG – 5 (T04) and unvaccinated control groups (T01) were not significantly different (*P* = 0.906) (Figure 3).

The scores for spontaneous coughing were summed per dog, and means were calculated. A significant treatment group effect was detected (*P* = 0.0001). Groups—T02 and T03 had significantly lower total scores than the negative control group (Table 2). Dogs in group—T04 had significantly higher scores than dogs in groups—T02 and T03. Treatment group—T01 and T04 were not statistically different. The mitigated fractions (MF) for spontaneous coughing were calculated. The MF is the probability that a vaccinated animal will have less severe disease across the observation period when compared to the negative control group. MF values in groups—T02 and T03, when compared to group—T01 were 0.7143 and 0.6964, respectively. Treatment group—T04 had a MF that was worse when compared to group—T01 (Table 2).

**Table 2:** Summary of spontaneous coughing: duration and totaled scores [results for euthanized dogs entered as the max observed score (2)].

**Treatment group**	**Duration (median number of days)**	**Scores totaled across days 43–54 (mean)**	**Mitigated fraction^††^ for total score (95% CI^‡^) (vs. T01)**
T01	11.00	13.29	–
T02	1.00^*^	1.75^*^	0.7143 (0.3618, 1.0000)
T03	1.00^*^	2.25^*^^a^	0.6964 (0.3251, 1.0000)
T04	7.00^†^	13.63^†^^b^	−0.2321 (−0.8350, 0.3708)
Overall treatment *P*-value	0.0003	0.0001	

†† *Mitigated fraction = the probability that a vaccinate will be less affected by challenge than a non-vaccinate. T01, Control Group. T02, Bronchicine CAe. T03, BbBG-7. T04, BbBG-5*.

* *vs. T01, P < 0.10*.

† *vs. T02, P < 0.10*.

‡ *Confidence interval*.

a, b *Among groups 2–4*.

*Values with no common letters are significantly different at P <0.10*.

**Figure 4:**
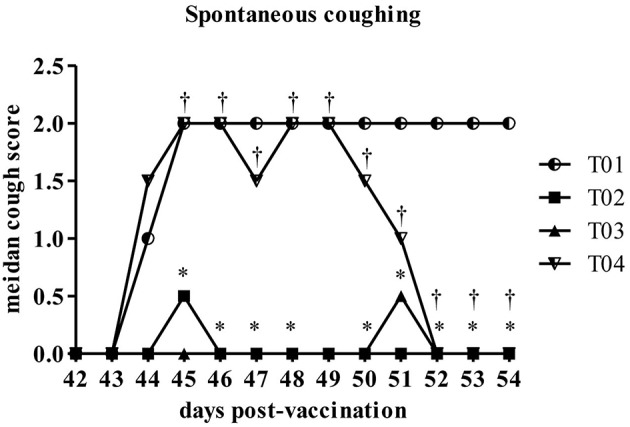
Summary of the spontaneous coughing scores by day post-challenge period [results for euthanized dogs entered as the max observed score (2)]. ^*^Within day vs. T01, *P* < 0.10. ^†^Within day vs. T02, *P* < 0.10. T01, PBS control. T02, Bronchicine CAe. T03, *B. bronchiseptica* Bacterial Ghosts (1 × 10^∧^7 cells/mL). T04, *B. bronchiseptica* Bacterial Ghosts (1 × 10^∧^5 cells/mL).

Spontaneous coughing scores were significantly lower in group—T02 on Days 45–48 and 50–54 as compared to the placebo control group—T01. Values in group—T03 were significantly lower than the placebo control group—T01 values on Days 44–48 and 50–54. When compared to the group—T02, values in group—T04 were significantly higher on Days 44–54 (Figure 4). T02 and T03 had significantly lower maximum scores compared to T01. There was no significant difference between T03 and the positive control T02. T04 had a higher maximum coughing score compared to T02 and T03.

**Figure 5:**
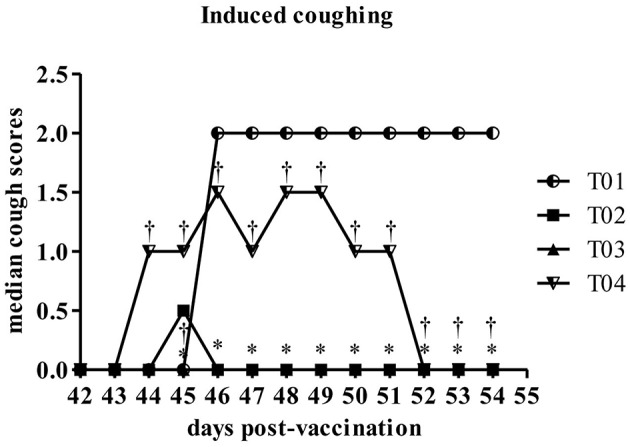
Summary of the coughing following laryngeal palpation scores by day post-challenge period [results for euthanized dogs entered as the max observed score (2)]. ^*^Within day vs. T01, *P* < 0.10. ^†^Within day vs. T02, *P* < 0.10. T01, PBS control. T02, Bronchicine CAe. T03, *B. bronchiseptica* Bacterial Ghosts (1 × 10^∧^7 cells/mL). T04, *B. bronchiseptica* Bacterial Ghosts (1 × 10^∧^5 cells/mL).

**Figure 6:**
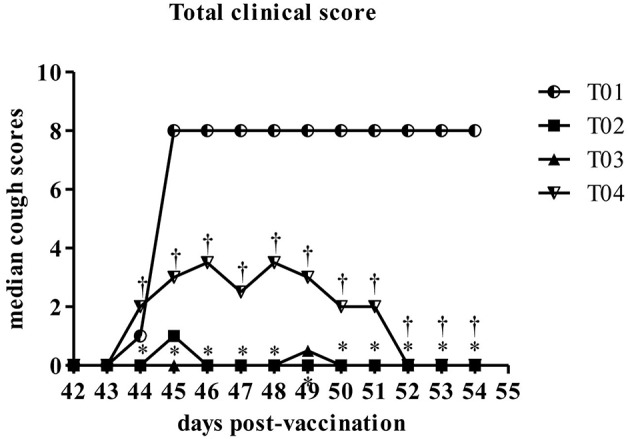
Summary of the total scores by day post-challenge period [results for euthanized dogs entered as the max observed score (8)]. ^*^Within day vs. T01, *P* < 0.10. ^†^Within day vs. T02, *P* < 0.10. T01, PBS control. T02, Bronchicine CAe. T03, *B. bronchiseptica* Bacterial Ghosts (1 × 10^∧^7 cells/mL). T04, *B. bronchiseptica* Bacterial Ghosts (1 × 10^∧^5 cells/mL).

Induced coughing scores were significantly lower in group—T02 and group—T03 when compared to values in control group—T01 or in group—T04 (Figure 5). Similarly total clinical scores (post challenge) were lower in group—T02 and T03 ranging between 1 and 0.5, respectively, as compared to values in group—T01 which was around 8 (max score) and for group—T04 in range of 2–3.5 (Figure 6). Other observed respiratory clinical signs were mucopurulent ocular and nasal discharge and sneezing. Only one dog had any recorded ocular or nasal discharge during the study. This dog was in treatment group—T04. Since serology and nasal shedding showed no discernable differences between any of the groups, the raw data is not shown here.

The new *B. bronchiseptica* BG vaccine tested in this study was found to be safe with a single small, transient injection site reaction after second vaccine dose administration. The challenge with virulent *Bordetella* strain was severe, as four dogs from placebo control and 3 dogs from BbBG – 5 group required euthanasia due to severity of respiratory disease. Even though it was an over-challenge, the BbBG – 7 vaccine was found to be efficacious against CIRD caused by *B. bronchiseptica*. No serious adverse event recorded following the administration of vaccines for the study. There were no behavioral changes (e.g., listlessness, depression) or any abnormal elevated temperatures following vaccination. Only two dogs showed mild injection site reaction, one in each group i.e., T02 (Bronchicine CAe) and T03 (BbBG – 7). These reactions occurred following the second vaccination. One animal belonging to PBS negative control group died due to complications not related to the vaccination study. Animals in negative control group showed severe signs of tracheobronchitis similarly, low dose BbBG – 5 vaccinated group also showed moderate to severe signs of tracheobronchitis. Dogs in vaccinated group—T02 (Bronchicine CAe) and T03 (BbBG – 7) did not show any sign of disease and were protected from tracheobronchitis.

## Discussion

This proof of concept study for testing of novel empty bacterial cell vaccine against virulent *B. bronchiseptica* strains demonstrated well and confirms the previous claims of efficacious *Bordetella* vaccines in dogs (17, 19, 20, 52–55). The challenge study did demonstrate efficacy of the BbBG – 7 vaccine against the severe disease, comparable to the existing *Bordetella* vaccine i.e., Bronchicine CAe which is a Cell, Antigen extract of *B. bronchise*ptica (56, 57). The s/c vaccination of the puppies with BbBG – 7 vaccine resulted in substantive decrease in coughing (most common sign of *Bordetella* infection) when compared to the negative control group—T01.

Further, the BbBG vaccine did demonstrate a dose dependent efficacy of the relatively lower dose (two log less of BG control) BbBG – 5 vaccine which was not effective to protect animals from clinical disease. The BbBG – 7 vaccine was used in concentrations up to 1 × 10^7^, in comparison to *Bronchicine CAe* which is a Cell, Antigen extract of 3 × 10^8^ *B. bronchiseptica* cells (Zoetis resource). There seems to be room for increasing the dose of BbBG vaccine in a more extended dose finding study.

In dog vaccination studies, routes of administration is highly debated topic and is often surrounded by controversies regarding the efficacy of parenteral, IN and oral *Bordetella* vaccination (18, 21, 56–60). In one of the early studies (57), it has been shown that the dogs vaccinated subcutaneously with acellular *Bordetella* (aB) vaccines showed higher serum concentration of *B. bronchiseptica* reactive IgG when compared to IN vaccinated group in seropositive dogs. In later studies (18, 59), this claim has been refuted and it was shown that the oral and IN route activates better salivary and serum immunoglobulin responses in seronegative dogs. In a recent study (60), it was shown that the previous history of infection (seropositive dogs) helps in a better and enhanced immune response in animals that are vaccinated subcutaneously with aB vaccine.

In the study presented, protection induced by parenteral administration of BbBG in seronegative dogs was explored. From previous BG vaccine studies (39, 61–64), it is well established that BGs can be effectively administered through, oral, rectal, mucosal, intranasal/aerogenic and parenteral routes, and conferred protective immunity in the vaccinated animals (mouse, rabbit, pigs, calves, etc.) against subsequent challenges. Since BGs are non-living cell envelops; it will also address the reservations of scientists who believe that IN administration of live attenuated *Bordetella* vaccines may put the owners of pets and veterinarians at risk of contracting *Bordetella* infection through shedding of bacterial droplets (3, 5, 7, 15, 65). A non-living BbBG vaccine is not able to induce infection in humans. Further BGs are shown to prevent bacterial colonization in a respiratory model. Pigs immunized either intramuscularly or aerogenically with *Actinobacillus pleuropneumoniae* BGs prevented bacterial colonization in lungs when compared to formalin inactivated *A. pleuropneumoniae* vaccine (38, 39, 61, 66). *B. pertussis* and *B. bronchiseptica* colonize the respiratory mucosa of humans and other mammals, respectively, via their fimbriae *fim2, fim3, fimX, and fimA* (67–70). Besides several studies on role of fimbriae in colonization of *B. bronchiseptica* and *B. pertussis* in respiratory mucosa, their precise role in pathogenesis of infection is yet to be concluded. It is due to this role that in some of the acellular pertussis (aP) vaccines *fim2* and *fim3* have been included (71). Fimbriae and pili are well preserved in BGs, and are part of vaccine preparations as shown with toxin co-regulated pili in *Vibrio cholera* BGs and fimbriae in *E. coli* BGs (37, 72). However, the presence of fimbriae in the BbBGs has not been investigated in current study and needs to be proved before any conclusions are extrapolated. In any case, a vaccine able to prevent bacterial colonization and conferring immunity against disease is superior to any other vaccine which is directed against the toxin alone which causes the disease. In current study we have not yet demonstrated that BbBGs prevent bacterial colonization as we have only explored subcutaneous route of immunization, other routes such as mucosal route may be required to demonstrate this.

Despite vigorous vaccination programs in developed countries, cases of pertussis have made a tremendous comeback. This comeback is mainly attributed to the use of aP vaccine. One of the hypotheses circulating among the scientific community is that the immunity from the aP vaccine diminishes faster as compared to the whole cell pertussis (Pw) vaccine (73). Secondly it is thought the aP vaccine provides protection against the disease but not colonization meaning that vaccine may not be effective at reducing the circulation of pertussis in the population or transmission to naïve individuals. BGs are shown to prevent bacterial colonization in a respiratory model. Further the route of immunization plays an important role in conferring full or partial immunity. Pigs immunized through the mucosal route, as oral (74) or as aerosols vaccine (61, 66), induce sterile immunity as demonstrated by the inability to isolate challenge bacteria from lungs and tonsils. Conversely an intramuscular immunization with *A. pleuropneumoniae* BGs protected pigs from clinical challenge and prevented bacterial colonization in lungs but the challenge bacteria could still be isolated from tonsils of vaccinated pigs (38, 39).

Acellular pertussis vaccines are usually less potent than Pw vaccines, with some exceptions (75, 76). The aP vaccines are alum based which promotes strong T helper type 2 (Th2), and T helper type 17 (Th17) antibody response, and lacks in producing cellular immunity linked to T helper type 1 (Th1) cells (77–82). A recent review (83) stresses for the need of Pertussis vaccine, that promotes Th1 mediate cellular immune response, which is thought to be more efficient in clearing *B. pertussis* from respiratory tract. In another study it is shown that the *Bordetella* colonization factor A, an outer membrane protein from *B. bronchiseptica* has strong adjuvant function and elicit both cellular and humoral immune response to heterologous and *B. pertussis* antigen (82). Similarly novel Toll-like receptor (TLR2)-activating lipoproteins from *B. pertussis* play an important role in activation of murine dendritic cells and macrophages and human mononuclear cells via TLR2 (84). Use of BG vaccines can circumvent the above requirements as they (BGs) are known for stimulating both cellular Th1 and humoral immune Th2 responses due to presence of essential structures on their cell surface including outer membrane proteins (85).

In study presented, a whole cell envelope BbBG vaccine, produced through a proprietary method was used. This BbBG vaccine exhibited a similar safety profile like, commercially available *Bordetella* vaccine (Bronchicine CAe). BGs vaccine technology can also be used for production of other *Bordetella* species (*B. pertussis, B. parapertussis*) vaccines, for prevention of whooping cough in humans. Whooping cough is considered to be reemerging disease by Center for Disease control (CDC) and is mostly linked to the use of aP vaccines (86). Once produced, these *B. pertussis* BGs will carry all essential surface structures that are necessary for triggering TLR mediated pathways, needed for efficient clearance of *B. pertussis* from respiratory mucosa.

There are between 370 and 1,500 per 100,000 cases of pertussis in adolescents and adults in the United States which is mostly attributed to the use of several less effective vaccines. Further, both immunity after infection and or after vaccination are not permanent. And can be an explanation for the increase in numbers of reported pertussis cases (87). The BG technology has an edge over other vaccines because of its ease of production and being stable in freeze-dried (several years), and in liquid formulation up-to several months. There is a need for efficient and affordable *Bordetella* vaccines especially in developing countries where *Bordetella* is endemic and is a major cause of morbidity and mortality (up to 90% of *B. pertussis* linked cases) in human populations (88). BGs might be able to fill this gap.

## Ethics Statement

The animal study was performed in accordance with the Guide for the Care and Use of Laboratory Animals (Eighth Edition, 2011) of National Research Council Academies. The National Academies Press, Washington DC, Title 9 Code of Federal Regulations, Part 103.3. The protocol was reviewed and approved by Institutional Animal Care and Use Committee of Ridglan Farms (no. BIOUS140044). This study was conducted to test efficacy of novel experimental biological products (vaccines) and did not represent an unnecessary duplication of research.

## Author Contributions

AM designed and together with JK developed the BbBG vaccine. MR produced study trial material. AM wrote this manuscript. WL did corrections and critical review of this paper. AF, AZ, and DW designed and supervised the animal study. PL together with AF did project coordination and project management.

### Conflict of Interest Statement

This study was conducted in collaboration with BIRD-C GmbH, Vienna, Austria and ELANCO Animal Health. AM, JK, MR, PL and WL are employed by BIRD-C. AF, AZ and DW are employed by ELANCO Animal Health, Greenfield, IN, USA. The authors declare that this study received funding from ELANCO Animal Health. ELANCO had the following involvement with the study: the funder financed and directed the animal studies, data collection and analysis. ELANCO and BIRD-C agreed to publish the study. BIRD-C GmbH produced the BbBG vaccine and covered the publication costs and is the propriety owner of BG platform technology.

## References

[1] Stefanelli P Mastrantonio P Hausman SZ Giuliano M Burns DL. Molecular characterization of two *Bordetella bronchiseptica* strains isolated from children with coughs. J Clin Microbiol. (1997) 35:1550–5. 9163480 10.1128/jcm.35.6.1550-1555.1997 PMC229785

[2] Dworkin MS Sullivan PS Buskin SE Harrington RD Olliffe J MacArthur RD. *Bordetella bronchiseptica* infection in human immunodeficiency virus-infected patients. Clin Infect Dis. (1999) 28:1095–9. 10.1086/514761 10452641

[3] Woolfrey BF Moody JA. Human infections associated with *Bordetella bronchiseptica*. Clin Microbiol Rev. (1991) 4:243–55. 10.1128/CMR.4.3.243 1889042 PMC358197

[4] Bauwens JE Spach DH Schacker TW Mustafa MM Bowden RA. *Bordetella bronchiseptica* pneumonia and bacteremia following bone marrow transplantation. J Clin Microbiol. (1992) 30:2474–5. 1401019 10.1128/jcm.30.9.2474-2475.1992 PMC265527

[5] Gueirard P Weber C Le Coustumier A Guiso N. Human *Bordetella bronchiseptica* infection related to contact with infected animals: persistence of bacteria in host. J Clin Microbiol. (1995) 33:2002–6. 7559937 10.1128/jcm.33.8.2002-2006.1995 PMC228324

[6] Bjornstad ON Harvill ET. Evolution and emergence of *Bordetella* in humans. Trends Microbiol. (2005) 13:355–9. 10.1016/j.tim.2005.06.007 15990312

[7] Gisel JJ Brumble LM Johnson MM. *Bordetella bronchiseptica* pneumonia in a kidney-pancreas transplant patient after exposure to recently vaccinated dogs. Transpl Infect Dis. (2010) 12:73–6. 10.1111/j.1399-3062.2009.00451.x 19874567

[8] Rampelotto RF Horner A Horner C Righi R Horner R. Pneumonia caused by *Bordetella bronchiseptica* in two HIV-positive patients. Sao Paulo Med J. (2016) 134:268–72. 10.1590/1516-3180.2015.02492701 27191248 PMC10496598

[9] Ferry NS A preliminary report of the bacterial findings in canine distemper. Am Vet Rev. (1910) 37:499–504.

[10] Ferry NS Etiology of canine distemper. J Infect Dis. (1911) 8:399–420. 10.1093/infdis/8.4.399

[11] Ferry NS *Bacillus bronchisepticus* (bronchicanis): the cause of distemper in dogs and a similar disease in other animals. Vet J. (1912) 68:376–91. 10.1016/S0372-5545(17)66038-3

[12] Ferry NS Bacteriology and control of acute infections in laboratory animals. J Pathol Bacteriol. (1913) 18:445–55. 10.1002/path.1700180143

[13] Smith T. Some bacteriological and environmental factors in the Pneumonias of lower animals with special reference to the Guinea-Pig. J Med Res. (1913) 29:291–324.295. 19972145 PMC2099764

[14] Ferry NS Canine distemper. Proc. Wis. Vet. Med. Assoc. (1917) 1917:80–88.

[15] Goodnow RA. Biology of *Bordetella bronchiseptica*. Microbiol Rev. (1980) 44:722–38. 7010115 10.1128/mr.44.4.722-738.1980 PMC373201

[16] Mann P Goebel E Barbarich J Pilione M Kennett M Harvill E. Use of a genetically defined double mutant strain of *Bordetella bronchiseptica* lacking adenylate cyclase and type III secretion as a live vaccine. Infect Immun. (2007) 75:3665–72. 10.1128/IAI.01648-06 17452472 PMC1932943

[17] McCandlish IA Thompson H Wright NG. Vaccination against *Bordetella bronchiseptica* infection in dogs using a heat-killed bacterial vaccine. Res Vet Sci. (1978) 25:45–50. 10.1016/S0034-5288(18)33007-8 705048

[18] Davis R Jayappa H Abdelmagid OY Armstrong R Sweeney D Lehr C. Comparison of the mucosal immune response in dogs vaccinated with either an intranasal avirulent live culture or a subcutaneous antigen extract vaccine of *Bordetella bronchiseptica*. Vet Ther. (2007) 8:32–40. Available online at: https://tinyurl.com/y6xaqu5s 17447223

[19] Shade FJ Goodnow RA. Intranasal immunization of dogs against *Bordetella bronchiseptica*-induced tracheobronchitis (kennel cough) with modified live-*Bordetella bronchiseptica* vaccine. Am J Vet Res. (1979) 40:1241–3. 525927

[20] Kontor EJ Wegrzyn RJ Goodnow RA. Canine infectious tracheobronchitis: effects of an intranasal live canine parainfluenza-*Bordetella bronchiseptica* vaccine on viral shedding and clinical tracheobronchitis (kennel cough). Am J Vet Res. (1981) 42:1694–8. 6275747

[21] Thomas JH Dana Parker S Alan Hassall J Chiang Y.-W Evaluation of efficacy of oral administration of *Bordetella bronchiseptica* intranasal vaccine when used to protect puppies from tracheobronchitis due to *B. bronchiseptica infection*. Intern J Appl Res Vet Med. (2011) 9:300–5. Available online at: https://tinyurl.com/y5an242r

[22] Anderton TL Maskell DJ Preston A. Ciliostasis is a key early event during colonization of canine tracheal tissue by *Bordetella bronchiseptica*. Microbiology. (2004) 150:2843–55. 10.1099/mic.0.27283-0 15347744

[23] Bemis DA Kennedy JR. An improved system for studying the effect of *Bordetella bronchiseptica* on the ciliary activity of canine tracheal epithelial cells. J Infect Dis. (1981) 144:349–57. 10.1093/infdis/144.4.349 7288215

[24] Ford RB Canine infectius tracheobronchitis. In: Greene C. E, editor. Infectious Diseases of Dog and Cat. Philadelphia, PA: Saunders; ELSVIER (2006). p. 54–61.

[25] Szostak MP Hensel A Eko FO Klein R Auer T Mader H. Bacterial ghosts: non-living candidate vaccines. J Biotechnol. (1996) 44:161–70. 10.1016/0168-1656(95)00123-9 8717400

[26] Lubitz W Witte A Eko FO Kamal M Jechlinger W Brand E. Extended recombinant bacterial ghost system. J Biotechnol. (1999) 73:261–73. 10.1016/S0168-1656(99)00144-3 10486935

[27] Jalava K Hensel A Szostak M Resch S Lubitz W. Bacterial ghosts as vaccine candidates for veterinary applications. J Control Release. (2002) 85:17–25. 10.1016/S0168-3659(02)00267-5 12480307

[28] Witte A Blasi U Halfmann G Szostak M Wanner G Lubitz W. Phi X174 protein E-mediated lysis of *Escherichia coli*. Biochimie. (1990) 72:191–200. 10.1016/0300-9084(90)90145-7 2143087

[29] Szostak M Lubitz W Recombinant bacterial ghosts as multivaccine vehicles. In: Chanock RM Ginsberg H Brown F Lerner R, editors. Vaccines 91: Modern Approaches to New Vaccines Including Prevention of AIDS. New York, NY: Cold Spring Harbor Laboratory Press (1991). p. 409–14.

[30] Mayr UB Kollera VJ Lubitz P Lubitz W Bacterial ghost as vaccine and drug delivery platforms. In: Roy S Colin H editors. Biotechnology Intelligence Unit. Austin, TX: Landes Bioscience (2008). p. 50–9.

[31] Lubitz P Mayr UB Lubitz W. Applications of bacterial ghosts in biomedicine. Adv Exp Med Biol. (2009) 655:159–70. 10.1007/978-1-4419-1132-2_12 20047041

[32] Muhammad A Champeimont J Mayr UB Lubitz W Kudela P. Bacterial ghosts as carriers of protein subunit and DNA-encoded antigens for vaccine applications. Expert Rev Vaccines. (2012) 11:97–116. 10.1586/erv.11.149 22149712

[33] Witte A Wanner G Blasi U Halfmann G Szostak M Lubitz W. Endogenous transmembrane tunnel formation mediated by phi X174 lysis protein E. J Bacteriol. (1990) 172:4109–14. 10.1128/jb.172.7.4109-4114.1990 2141836 PMC213400

[34] Huter V Szostak MP Gampfer J Prethaler S Wanner G Gabor F. Bacterial ghosts as drug carrier and targeting vehicles. J Control Release. (1999) 61:51–63. 10.1016/S0168-3659(99)00099-1 10469902

[35] Paukner S Kohl G Jalava K Lubitz W. Sealed bacterial ghosts–novel targeting vehicles for advanced drug delivery of water-soluble substances. J Drug Target. (2003) 11:151–61. 10.1080/10611860310001593366 13129825

[36] Witte A Wanner G Sulzner M Lubitz W. Dynamics of PhiX174 protein E-mediated lysis of Escherichia coli. Arch Microbiol. (1992) 157:381–8. 10.1007/BF00248685 1534215

[37] Eko FO Mayr UB Attridge SR Lubitz W. Characterization and immunogenicity of *Vibrio cholerae* ghosts expressing toxin-coregulated pili. J Biotechnol. (2000) 83:115–23. 10.1016/S0168-1656(00)00315-1 11000467

[38] Hensel A Huter V Katinger A Raza P Strnistschie C Roesler U. Intramuscular immunization with genetically inactivated (ghosts) Actinobacillus pleuropneumoniae serotype 9 protects pigs against homologous aerosol challenge and prevents carrier state. Vaccine. (2000) 18:2945–55. 10.1016/S0264-410X(00)00107-9 10825595

[39] Huter V Hensel A Brand E Lubitz W. Improved protection against lung colonization by *Actinobacillus pleuropneumoniae* ghosts: characterization of a genetically inactivated vaccine. J Biotechnol. (2000) 83:161–72. 10.1016/S0168-1656(00)00310-2 11000472

[40] Riedmann EM Kyd JM Cripps AW Lubitz W. Bacterial ghosts as adjuvant particles. Expert Rev Vaccines. (2007) 6:241–53. 10.1586/14760584.6.2.241 17408373

[41] Krasko JA Zilionyte K Darinskas A Strioga M Rjabceva S Zalutsky I. Bacterial ghosts as adjuvants in syngeneic tumour cell lysate-based anticancer vaccination in a murine lung carcinoma model. Oncol Rep. (2017) 37:171–8. 10.3892/or.2016.5252 27878261

[42] Michalek J Hezova R Turanek-Knotigova P Gabkova J Strioga M Lubitz W. Oncolysate-loaded *Escherichia coli* bacterial ghosts enhance the stimulatory capacity of human dendritic cells. Cancer Immunol Immunother. (2017) 66:149–59. 10.1007/s00262-016-1932-4 27864613 PMC11029152

[43] Langemann T Koller VJ Muhammad A Kudela P Mayr UB Lubitz W. The bacterial ghost platform system: production and applications. Bioeng Bugs. (2010) 1:326–36. 10.4161/bbug.1.5.12540 21326832 PMC3037582

[44] Kovach ME Elzer PH Hill DS Robertson GT Farris MA Roop RMII. Four new derivatives of the broad-host-range cloning vector pBBR1MCS, carrying different antibiotic-resistance cassettes. Gene. (1995) 166:175–6. 10.1016/0378-1119(95)00584-1 8529885

[45] Miller JF Dower WJ Tompkins LS. High-voltage electroporation of bacteria: genetic transformation of *Campylobacter jejuni* with plasmid DNA. Proc Natl Acad Sci USA. (1988) 85:856–60. 10.1073/pnas.85.3.856 3277182 PMC279654

[46] Mobley DM Chengappa MM Kadel WL Stuart JG. Effect of pH, temperature and media on acid and alkaline phosphatase activity in clinical and nonclinical isolates of *Bordetella bronchiseptica*. Can J Comp Med. (1984) 48:175–8. 6722645 PMC1236033

[47] EMA General Requirements for the Production and Control of Inactivated Mammalian Bacterial and Viral Vaccines for Veterinary Use. E. M. Association. (1999).

[48] Dees C Fountain MW Panangala VS Swango LJ Schultz RD. An ELISA test to detect antibody to *Bordetella bronchiseptica*. Vet Immunol Immunopathol. (1982) 3:539–45. 10.1016/0165-2427(82)90037-X 7179721

[49] Scott-Garrard MM Chiang YW David F. Comparative onset of immunity of oral and intranasal vaccines against challenge with *Bordetella bronchiseptica*. Vet Rec Open. (2018) 5:e000285. 10.1136/vetreco-2018-000285 30167313 PMC6109801

[50] Smith IM Baskerville AJ. A selective medium facilitating the isolation and recognition of *Bordetella bronchiseptica* in pigs. Res Vet Sci. (1979) 27:187–92. 10.1016/S0034-5288(18)32826-1 42958

[51] Barry AL Clinical specimens for microbiological examination. In: Haeprich PD, editor. Infectious Diseases a Guide to the Understanding and Management of Infectious Processes. New York, NY: Harper and Row (1972). p. 103–7.

[52] McCandlish IA Thompson H Wright NG. Vaccination against canine bordetellosis using an aluminum hydroxide adjuvant vaccine. Res Vet Sci. (1978) 25:51–7. 10.1016/S0034-5288(18)33008-X 705049

[53] Bey RF Shade FJ Goodnow RA Johnson RC. Intranasal vaccination of dogs with liver avirulent *Bordetella bronchiseptica*: correlation of serum agglutination titer and the formation of secretory IgA with protection against experimentally induced infectious tracheobronchitis. Am J Vet Res. (1981) 42:1130–2. 7271029

[54] Glickman LT Appel MJ. Intranasal vaccine trial for canine infectious tracheobronchitis (kennel cough). Lab Anim Sci. (1981) 31:397–9. 6273648

[55] Shade FJ Rapp VJ Studies of a bacterin incorporating an extracted *Bordetella bronchiseptica* antigen for controlling canine bordetellosis. Vet Med Small Anim Clin. (1982) 77:1635–9.

[56] Ellis JA Haines DM West KH Burr JH Dayton A Townsend HG. Effect of vaccination on experimental infection with *Bordetella bronchiseptica* in dogs. J Am Vet Med Assoc. (2001) 218:367–75. 10.2460/javma.2001.218.367 11201562

[57] Ellis JA Krakowka GS Dayton AD Konoby C. Comparative efficacy of an injectable vaccine and an intranasal vaccine in stimulating *Bordetella bronchiseptica*-reactive antibody responses in seropositive dogs. J Am Vet Med Assoc. (2002) 220:43–8. 10.2460/javma.2002.220.43 12680446

[58] Gore T Headley M Laris R Bergman JG Sutton D Horspool LJ. Intranasal kennel cough vaccine protecting dogs from experimental *Bordetella bronchiseptica* challenge within 72 hours. Vet Rec. (2005) 156:482–3. 10.1136/vr.156.15.482 15828745

[59] Larson LJ Theil BE Sharp P Schultz RD A comparative study of protective immunity provided by oral, intranasal and parenteral canine *Bordetella bronchiseptica* vaccines. Intern J Appl Res Vet Med. (2013) 11:153–60. Available online at: https://tinyurl.com/y4uegzwn

[60] Ellis J Rhodes C Lacoste S Krakowka S. Antibody responses to *Bordetella bronchiseptica* in vaccinated and infected dogs. Can Vet J. (2014) 55:857–64. 25183893 PMC4137927

[61] Katinger A Lubitz W Szostak MP Stadler M Klein R Indra A. Pigs aerogenously immunized with genetically inactivated (ghosts) or irradiated *Actinobacillus pleuropneumoniae* are protected against a homologous aerosol challenge despite differing in pulmonary cellular and antibody responses. J Biotechnol. (1999) 73:251–60. 10.1016/S0168-1656(99)00143-1 10486934

[62] Mayr UB Haller C Haidinger W Atrasheuskaya A Bukin E Lubitz W. Bacterial ghosts as an oral vaccine: a single dose of *Escherichia coli* O157:H7 bacterial ghosts protects mice against lethal challenge. Infect Immun. (2005) 73:4810–7. 10.1128/IAI.73.8.4810-4817.2005 16040994 PMC1201255

[63] Walcher P Cui X Arrow JA Scobie S Molinia FC Cowan PE. Bacterial ghosts as a delivery system for zona pellucida-2 fertility control vaccines for brushtail possums (*Trichosurus vulpecula*). Vaccine. (2008) 26:6832–8. 10.1016/j.vaccine.2008.09.088 18948157

[64] Mayr UB Kudela P Atrasheuskaya A Bukin E Ignatyev G Lubitz W. Rectal single dose immunization of mice with *Escherichia coli* O157:H7 bacterial ghosts induces efficient humoral and cellular immune responses, and protects against the lethal heterologous challenge. Microb Biotechnol. (2012) 5:283–94. 10.1111/j.1751-7915.2011.00316.x 22103353 PMC3815788

[65] de la Torre MJ de la Fuente CG de Alegria CR Del Molino CP Aguero J Martinez-Martinez L. Recurrent respiratory infection caused by *Bordetella bronchiseptica* in an immunocompetent infant. Pediatr Infect Dis J. (2012) 31:981–3. 10.1097/INF.0b013e31825d2e84 22572751

[66] Hensel A van Leengoed LA Szostak M Windt H Weissenbock H Stockhofe-Zurwieden N. Induction of protective immunity by aerosol or oral application of candidate vaccines in a dose-controlled pig aerosol infection model. J Biotechnol. (1996) 44:171–81. 10.1016/0168-1656(95)00150-6 8717401

[67] Livey I Duggleby CJ Robinson A. Cloning and nucleotide sequence analysis of the serotype 2 fimbrial subunit gene of *Bordetella pertussis*. Mol Microbiol. (1987) 1:203–9. 10.1111/j.1365-2958.1987.tb00513.x 2897065

[68] Pedroni P Riboli B de Ferra F Grandi G Toma S Arico B. Cloning of a novel pilin-like gene from *Bordetella pertussis*: homology to the fim2 gene. Mol Microbiol. (1988) 2:539–43. 10.1111/j.1365-2958.1988.tb00061.x 2902506

[69] Cuzzoni A Pedroni P Riboli B Grandi G de Ferra F. Nucleotide sequence of the fim3 gene from *Bordetella pertussis* and homology to fim2 and fimX gene products. Nucleic Acids Res. (1990) 18:1640. 10.1093/nar/18.6.1640 1970172 PMC330546

[70] Boschwitz JS van der Heide HG Mooi FR Relman DA. *Bordetella bronchiseptica* expresses the fimbrial structural subunit gene fimA. J Bacteriol. (1997) 179:7882–5. 10.1128/jb.179.24.7882-7885.1997 9401052 PMC179756

[71] Gustafsson L Hallander HO Olin P Reizenstein E Storsaeter J. A controlled trial of a two-component acellular, a five-component acellular, and a whole-cell pertussis vaccine. N Engl J Med. (1996) 334:349–55. 10.1056/NEJM199602083340602 8538705

[72] Montanaro J Inic-Kanada A Ladurner A Stein E Belij S Bintner N. *Escherichia coli* Nissle 1917 bacterial ghosts retain crucial surface properties, and express chlamydial antigen: an imaging study of a delivery system for the ocular surface. Drug Des Devel Ther. (2015) 9:3741–54. 10.2147/DDDT.S84370 26229437 PMC4516183

[73] Witt MA Katz PH Witt DJ. Unexpectedly limited durability of immunity following acellular pertussis vaccination in preadolescents in a North American outbreak. Clin Infect Dis. (2012) 54:1730–5. 10.1093/cid/cis287 22423127

[74] Hensel A Stockhofe-Zurwieden N Petzoldt K Lubitz W. Oral immunization of pigs with viable or inactivated *Actinobacillus pleuropneumoniae* serotype 9 induces pulmonary and systemic antibodies and protects against homologous aerosol challenge. Infect Immun. (1995) 63:3048–53. 10.1016/0378-1135(95)00106-K 7622229 PMC173415

[75] Storsaeter J Wolter J Locht C Pertussis vaccines. In: Locht C, editor. Bordetella Molecular Microbiology. Norfolk: Horizon Biosciences (2007). p. 245–288.

[76] Klein NP Bartlett J Rowhani-Rahbar A Fireman B Baxter R. Waning protection after fifth dose of acellular pertussis vaccine in children. N Engl J Med. (2012) 367:1012–9. 10.1056/NEJMoa1200850 22970945

[77] Redhead K Watkins J Barnard A Mills KH. Effective immunization against *Bordetella pertussis* respiratory infection in mice is dependent on induction of cell-mediated immunity. Infect Immun. (1993) 61:3190–8. 8335349 10.1128/iai.61.8.3190-3198.1993 PMC280987

[78] Ausiello CM Urbani F la Sala A Lande R Cassone A Vaccine- and antigen-dependent type 1 and type 2 cytokine induction after primary vaccination of infants with whole-cell or acellular pertussis vaccines. Infect Immun. (1997) 65:2168–74. 9169747 10.1128/iai.65.6.2168-2174.1997 PMC175299

[79] Ryan M Murphy G Ryan E Nilsson L Shackley F Gothefors L. Distinct T-cell subtypes induced with whole cell and acellular pertussis vaccines in children. Immunology. (1998) 93:1–10. 10.1046/j.1365-2567.1998.00401.x 9536112 PMC1364099

[80] Ryan EJ Nilsson L Kjellman N Gothefors L Mills KH Booster immunization of children with an acellular pertussis vaccine enhances Th2 cytokine production and serum IgE responses against pertussis toxin but not against common allergens. Clin Exp Immunol. (2000) 121:193–200. 10.1046/j.1365-2249.2000.01306.x 10931131 PMC1905694

[81] Ross PJ Sutton CE Higgins S Allen AC Walsh K Misiak A. Relative contribution of Th1 and Th17 cells in adaptive immunity to *Bordetella pertussis*: towards the rational design of an improved acellular pertussis vaccine. PLoS Pathog. (2013) 9:e1003264. 10.1371/journal.ppat.1003264 23592988 PMC3617212

[82] Jennings-Gee J Quataert S Ganguly T D'Agostino RJr Deora R Dubey P. The adjuvant *Bordetella* Colonization Factor A attenuates alum-induced Th2 responses and enhances *Bordetella pertussis* clearance from mouse lungs. Infect Immun. (2018) 86:e00935-17. 10.1128/IAI.00935-17 29531137 PMC5964531

[83] Higgs R Higgins SC Ross PJ Mills KH. Immunity to the respiratory pathogen *Bordetella pertussis*. Mucosal Immunol. (2012) 5:485–500. 10.1038/mi.2012.54 22718262

[84] Dunne A Mielke LA Allen AC Sutton CE Higgs R Cunningham CC. A novel TLR2 agonist from *Bordetella pertussis* is a potent adjuvant that promotes protective immunity with an acellular pertussis vaccine. Mucosal Immunol. (2015) 8:607–17. 10.1038/mi.2014.93 25315966

[85] Ebensen T Paukner S Link C Kudela P de Domenico C Lubitz W. Bacterial ghosts are an efficient delivery system for DNA vaccines. J Immunol. (2004) 172:6858–65. 10.4049/jimmunol.172.11.6858 15153504

[86] CDC PErtussis—united states, 1997–2000. JAMA. (2002) 287:977–9. 10.1001/jama.287.8.977 11871441

[87] Cherry JD. The epidemiology of pertussis: a comparison of the epidemiology of the disease pertussis with the epidemiology of *Bordetella pertussis* infection. Pediatrics. (2005) 115:1422–7. 10.1542/peds.2004-2648 15867059

[88] Locht C. A common vaccination strategy to solve unsolved problems of tuberculosis and pertussis? Microbes Infect. (2008) 10:1051–6. 10.1016/j.micinf.2008.07.008 18672086

