# Corrigendum: Subcutaneous Immunization of Dogs With *Bordetella bronchiseptica* Bacterial Ghost Vaccine

**DOI:** 10.3389/fimmu.2020.00193

**Published:** 2020-02-12

**Authors:** Abbas Muhammad, Johannes Kassmannhuber, Mascha Rauscher, Alaric A. Falcon, David W. Wheeler, Alan A. Zhang, Petra Lubitz, Werner Lubitz

**Affiliations:** ^1^BIRD-C GmbH & Co KG, Vienna, Austria; ^2^Centre of Molecular Biology, University of Vienna, Vienna, Austria; ^3^ELANCO Animal Health, Greenfield, IN, United States

**Keywords:** *Bordetella*, Bacterial Ghosts (BGs), whooping cough, immunization (vaccination), dog model

An author name was incorrectly spelled as **Mascha Raucher**. The correct spelling is **Mascha Rauscher**.

The authors apologize for this error and state that this does not change the scientific conclusions of the article in any way. The original article has been updated.

